# Advances in systemic immune inflammatory indices in non-small cell lung cancer: A review

**DOI:** 10.1097/MD.0000000000037967

**Published:** 2024-05-03

**Authors:** Kai-Yun Mao, Yuan-Chao Cao, Mao-Yan Si, Ding-yu Rao, Liang Gu, Zhi-Xian Tang, Shen-yu Zhu

**Affiliations:** a First Clinical Medical College, Gannan Medical University, Ganzhou, China; b Department of Cardiothoracic Surgery, The First Affiliated Hospital of Gannan Medical University, Ganzhou, China.

**Keywords:** cancer, inflammation, non-small cell lung cancer (NSCLC), systemic immunoinflammatory index (SII)

## Abstract

Lung cancer is one of the most prevalent cancers globally, with non-small cell lung cancers constituting the majority. These cancers have a high incidence and mortality rate. In recent years, a growing body of research has demonstrated the intricate link between inflammation and cancer, highlighting that inflammation and cancer are inextricably linked and that inflammation plays a pivotal role in cancer development, progression, and prognosis of cancer. The Systemic Immunoinflammatory Index (SII), comprising neutrophil, lymphocyte, and platelet counts, is a more comprehensive indicator of the host’s systemic inflammation and immune status than a single inflammatory index. It is widely used in clinical practice due to its cost-effectiveness, simplicity, noninvasiveness, and ease of acquisition. This paper reviews the impact of SII on the development, progression, and prognosis of non-small cell lung cancer.

## 1. Introduction

According to the latest WHO GLOBOCAN 2020 global cancer statistics, the top 5 new cancer cancers in 2020 are breast, lung, colon, prostate, and stomach. Among them, lung cancer is the leading cause of cancer deaths, followed by colorectal, liver, stomach, and female breast cancers, accounting for 6.9% of all cancer deaths.^[1]^ Lung cancer poses a significant threat to human life and health, accounting for approximately one-fifth of all cancer deaths worldwide in 2020. It includes small cell lung cancer (SCLC) and non-small cell lung cancer (NSCLC), with NSCLC accounting for approximately 85% of all cases.^[2]^ NSCLC is mainly represented by adenocarcinoma of the lung (ADC), squamous cell carcinoma (SCC), and large-cell lung cancer. Unfortunately, due to the lack of a specific clinical presentations in patients with early-stage NSCLC, most patients with NSCLC are diagnosed at an advanced stage, missing the optimal treatment window. Despite advancements in diagnostic tumor biomarkers and therapeutic approaches such as surgery, chemotherapy, radiotherapy, targeted therapy, and immunotherapy, the 5-year overall survival rate for lung cancer remains poor.^[3]^ The systemic immunoinflammatory index (SII) is a readily available inflammatory index in peripheral blood, which is cost-effective and simple. This paper reviews the impact of SII on the development, progression, and prognosis of lung cancer.

## 2. The relationship between tumors and inflammation

**Figure 1.**
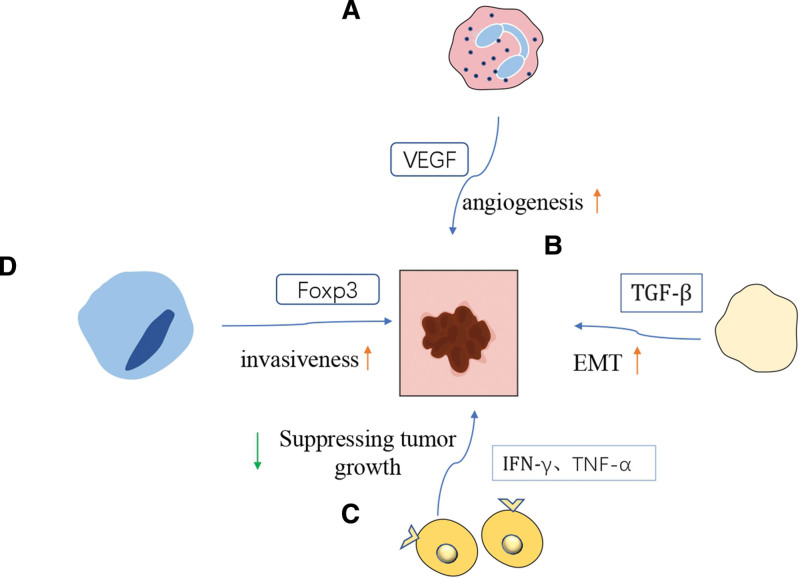
(A) Neutrophils support pro-angiogenic conversion in cancer by releasing VEGF and other pro-angiogenic factors. (B) Platelet-derived TGF-β can synergistically activate the TGF-β/Smad and NF-κB pathways in cancer cells to promote epithelial-mesenchymal transition (EMT) in vivo. (C) CD8 T cells and CD4 T cells may cooperate to inhibit cancer progression. (D) Foxp3 overexpression in Treg cells enhances the viability and aggressiveness of lung cancer cells. EMT = epithelial-mesenchymal transition, NF-κB = nuclear factor-kappa B.

The relationship between tumors and inflammation has been recognized since the 19th century when Rudolf Virchow proposed that tumors could arise from chronic inflammation (As shown in the Fig. 1).^[4–6]^ In recent years, extensive research has revealed the intricate connection between tumors and inflammation.^[7–10]^ It has been found that the molecular and cellular mechanisms linking inflammation and cancer involve 2 pathways. The intrinsic pathway is initiated by genetic events that lead to tumor formation and subsequently activate inflammation-related programs, shaping the inflammatory microenvironment. The extrinsic pathway, on the other hand, involves the promotion of cancer development by inflammatory conditions.^[11]^ Studies by Candido et al have demonstrated the role of the inflammatory response in tumor progression, invasion, and metastasis. This response is mediated through the regulation of transcription factor NF-kappaB and signaling molecule STAT3, which contribute to tumor angiogenesis and inhibit anticancer activity.^[9]^

In recent years, several studies have highlighted the significance of inflammatory biomarkers, including C-reactive protein, platelets, neutrophils, platelet-to-lymphocyte ratio (PLR), neutrophil-to-lymphocyte ratio (NLR), and lymphocytes, in cancer.^[12]^ The Systemic Immunoinflammatory Index (SII), which was first proposed by Hu et al in 2014, is an index calculated based on peripheral blood neutrophil (N), lymphocyte (L), and platelet (P) counts using the formula SII = (P × N)/L. SII has been shown to be a robust prognostic indicator of poor outcomes in patients with hepatocellular carcinoma.^[13]^

### 2.1. Mechanism of action of systemic immunoinflammatory index in non-small cell lung cancer

To date, the mechanism by which SII affects NSCLC is not clearly understood for the time being, but there are several theories to support this phenomenon, which are as follows:

#### 2.1.1. Neutrophils and non-small cell lung cancer

Neutrophils, as the most abundant White blood cells in peripheral blood, play a crucial role in the first-line of innate immune defense system. Research by SZCZERBA et al has shown that neutrophils not only provide barrier protection to tumors but also to influence the gene expression profile of tumor cells. They secrete cytokines such as interleukin-1 and IL-6, leading to enhanced proliferation characteristics in tumor cells, thereby promoting metastasis and reducing host survival.^[14]^ Huang et al have demonstrated that neutrophils could release inflammatory factors, including neutrophil elastase and matrix metalloproteinase 9, which contribute to tumor proliferation and metastasis.^[15]^ Furthermore, neutrophils are involved in promoting angiogenesis in cancer by releasing vascular endothelial growth factor (VEGF) and other pro-angiogenic factors.^[16]^

Another important factor associated with neutrophils is serum amyloid A (SAA), which serves as a potent neutrophil chemotactic factor for neutrophils and promotes their recruitment to the lung. Studies have observed a positive correlation between the number of neutrophils and SAA expression in peripheral lung cancer resection samples, and lung cancer patients exhibit significantly elevated levels of SAA in their serum and plasma.^[17]^

Neutrophils also play a role in tumor progression through the generation of reactive oxygen species (ROS) in the tumor microenvironment. High levels of ROS contribute to various aspects of tumor progression, including inflammation, cell proliferation, and cell survival. Additionally, ROS can stimulate the activity of nuclear factor-kappa B (NF-κB), which is known to be involved in different stages of cancer development and progression. NF-κB activation has the ability to transform otherwise normal cells into malignant cells.^[18]^

#### 2.1.2. Lymphocytes and non-small cell lung cancer

Lymphocytes play a crucial role in immune defense, immune surveillance, and antitumor immune responses.^[19]^ They are the core of the immune response and are usually classified into various subtypes, including T lymphocytes, B lymphocytes, and natural killer cells. Lymphocytes exert their anticancer effects through promoting the secretion of relevant cytokines and chemokines, such as interferon-gamma (IFN-γ) and tumor necrosis factor-alpha (TNF-α), which indirectly contribute to the antitumor immune response and directly induce tumor cell killing.^[20]^

In the context of non-small cell lung cancer (NSCLC), CD8 T cells and CD4 T cells have been found to cooperate in inhibiting cancer progression, suggesting their potential as indicators for adjuvant therapy in patients with poor prognosis.^[21]^ Regulatory T cells (Tregs), a subset of lymphocytes, have been associated with poor prognosis in lung adenocarcinoma.^[22,23]^ It has also been observed that overexpression of Foxp3 in Treg cells enhances the viability and aggressiveness of lung cancer cells.^[24–26]^ Manipulating the number of Treg cells has been shown to affect the tumor-killing capacity of immune cells, with a decrease in Treg cells resulting in increased immune cell-mediated tumor-killing.^[27,28]^

The number and subtype of lymphocytes have been suggested to be closely related to the survival rate of cancer patients.^[29–31]^ However, there is still no definite conclusion and a lot of research needs to be done to further research is needed to establish a definitive conclusion in this regard. More studies are required to confirm and understand the intricate relationship between lymphocytes and cancer prognosis.

#### 2.1.3. Platelets and non-small cell lung cancer

Platelets and coagulation factors have been found to play a significant role in the tumor microenvironment. It was first suggested by Armand Trousseau in 1865 that increased blood coagulability is associated with cancer cells.^[32]^ Tumor cells can activate platelets through the production of tissue factor and the expression of particulate tissue factor, matrix metalloproteinase, and thrombin, leading to thrombosis promotion.^[33,34]^ The interaction between platelets and cancer cells has been shown to have various effects on tumor progression.^[35]^

Studies have demonstrated that platelet release can inhibit the expression of Krüppel-like factor 6 (KLF6), promoting malignant proliferation.^[36]^ Platelet-cancer cell interactions and platelet-derived transforming growth factor-beta (TGF-β) can activate the TGF-β/Smad and NF-κB pathways in cancer cells, promoting epithelial-mesenchymal transition (EMT) and enhancing cancer cell adhesion and metastatic potential.^[37]^ Elevated platelet counts have been associated with poor prognosis in lung cancer patients, as concluded from a meta-analysis of 40 studies.^[38]^

Moreover, the interaction between platelets and oncoprotein, mediated by C-type lectin-like immunoreceptor 2 (CLEC-2),^[39]^ has been implicated in tumor growth, EMT, invasion, metastasis, and cancer-induced thrombosis.^[40]^ CLEC-2, identified as a platelet receptor, plays a functional role in tumor progression and metastasis, with platelet CLEC-2 mediating these processes.^[41]^

### 2.2 Application of systemic immunoinflammatory index in non-small cell lung cancer

#### 2.2.1. T, N, M staging

The TNM stage of lung cancer is a crucial factor in guiding the treatment and predicting the prognosis of non-small cell lung cancer (NSCLC).^[42]^ However, there is a need for effective preoperative tests to predict the pathological staging. The results of NSCLC. Several studies have investigated the association between the systemic immunoinflammatory index (SII) and TNM staging in NSCLC patients.^[13,43]^

In a retrospective study by Yang et al involving 233 NSCLC patients, higher the preoperative SII values were found to be associated with advanced T and N stages. Patients with high SII values and NSCLC were more likely to have advanced T-stage and lymph node metastases.^[44]^ The study also reported significant differences in SII values between the NSCLC group and the healthy control group and the NSCLC group, with significantly higher SII values observed in the NSCLC group.^[44]^ These findings suggested that changes in SII values may indicate tumor progression.^[45,46]^

Another retrospective study by Gao et al involving 400 NSCLC patients showed that high SII was significantly associated with advanced T-stage and positive lymph node metastasis.^[47,48]^ Similarly, Tomita et al conducted a study on 471 NSCLC patients and found that low SII was significantly associated with the lower pathological TNM stage.^[49,50]^

#### 2.2.2. Prognosis and survival

Several studies have examined the prognostic value of the SII in patients with non-small cell lung cancer (NSCLC) and have consistently reported its association with poorer survival outcomes. In a retrospective analysis of 3984 NSCLC patients by Fu et al, it was found that high SII was associated with poorer recurrence-free survival (*P* < .001) and overall survival (OS). SII was identified as an independent risk factor for worsening recurrence-free survival (*P* = .038) and OS. Further subgroup analysis showed that the prognostic value of SII was particularly evident in patients with stage I disease, solid nodes, or adenocarcinoma.^[51]^ Guo et al, in their analysis of medical records from 2018 NSCLC patients, Guo et al found that preoperative SII was an independent prognostic biomarker for overall survival (OS) in surgically resected NSCLC patients. They also observed that higher SII values may be associated with tumor angiogenesis, invasion, and metastasis, leading to poor survival. Additionally, they concluded that SII had better prognostic power compared to other inflammatory markers such as neutrophil-to-lymphocyte ratio (NLR) and PLR.^[52,53]^ Another study reported that higher SII values were associated with lower progression-free survival (*P* < .001) and lower overall survival in NSCLC patients. Multivariate Cox analysis confirmed SII as an independent prognostic factor affecting overall survival (*P* = .010; risk ratio = 2.824) and progression-free survival.^[54]^ Huang et al conducted a study involving 8 studies and a total of 752 NSCLC patients, and they found that patients with NSCLC with high levels of SII had poorer overall survival compared to those with low levels of SII.^[55]^

#### 2.2.3. Adjuvant radiotherapy and chemotherapy

Keit et al, in their analysis of 125 patients who received concurrent radiotherapy, observed that patients with low SII had significantly longer median progression-free survival (PFS) and overall survival (OS) compared to those with high SII. The median PFS and OS for patients with low SII were 4.8 months and 27.2 months, respectively, whereas those with high SII had a median PFS of 1.0 month and a median OS of 13.1 months. Additionally, the 5-year OS rate and PFS were higher in the low SII group (33.8% and 18.4%, respectively) compared to the high SII group (5.8% and 4.7%, respectively). These findings suggest that low SII is significantly associated with improved OS and PFS in patients undergoing radiotherapy.^[56]^ Delikgoz Soykut et al analyzed 217 patients and found that the low SII group exhibited better radiosensitivity, radiotherapy efficiency, and overall prognosis compared to the high SII group.^[57]^

Similarly, Tong et al, in a study involving 217 patients receiving concurrent radiotherapy, reported that patients with low SII had a significantly higher response rate to treatment compared to those with high SII. This association may be due to the link between high SII and radiotherapy resistance.^[58]^

#### 2.2.4. Metastases

Xu et al conducted a study involving 234 patients with pathologically confirmed bone metastases from non-small cell lung cancer (NSCLC).^[46]^ They found a significant association between pretreatment SII values and the number of distant metastases. Patients with higher SII values had a higher number of distant metastases, indicating a potential role of SII in predicting metastatic spread.^[59]^ In another retrospective study by Wang et al, patients with brain metastases from NSCLC were analyzed. Low SII levels were associated with inhibited tumor growth and metastasis, leading to a better prognosis when treated with adjuvant chemotherapy.^[60]^

#### 2.2.5. Immune checkpoint inhibitors & systemic immunoinflammatory index

Immune checkpoint inhibitors (ICIs) have substantially improved the prognosis of many patients with advanced cancer. However, some patients still do not benefit from ICIs. Therefore, determining indicators that can identify patients who may benefit from ICIs is essential. As a noninvasive, convenient and inexpensive clinical indicator, the systemic immune–inflammation index is expected to solve the aforementioned issue. The study found that ICIs treatments significantly changed the levels of SII, NLR, PLR, LCR and LMR in NPC patients treated with immunotherapy. A lower baseline SII and a higher baseline LMR, and a reduction in SII and an elevation in LMR after immunotherapy are favorable factors for predicting survival among advanced NPC patients.^[61]^ In addition, the NLR and SII are associated with OS and thus prognosis in a large prospective cohort of patients with different tumor types treated with ICIs. This is consistent whether the patient is being treated in a first-line setting or beyond.^[62]^ In conclusion, SII can be used as an effective biomarker and easily accessible prognostic marker for patients undergoing ICIs.

## 3. Discussion

In summary, SII, as a readily available inflammatory index in peripheral blood, SII holds promise in predicting patient prognosis, guiding individualized treatment plans, reducing tumor recurrence and metastasis, and improving the quality of life for NSCLC patients. While numerous studies have demonstrated the correlation of SII correlates with TNM stage, prognosis, survival rate, and adjuvant radiotherapy in NSCLC, its use in histological staging has not been extensively reported. Further exploration through large sample sizes and multicenter prospective studies is needed to establish appropriate and standardized threshold values for SII, enabling its optimal clinical application and providing a theoretical basis for diagnosing and treating NSCLC.

## Acknowledgments

We are deeply grateful for the guidance and assistance provided by the Key Laboratory of Cardiology at Gannan Medical University.

## Author contributions

**Conceptualization:** KaiYun Mao, Shen-yu Zhu.

**Data curation:** KaiYun Mao, Mao-Yan Si, Shen-yu Zhu.

**Formal analysis:** KaiYun Mao, Yuan-Chao Cao, Liang Gu, Zhi-Xian Tang, Shen-yu Zhu.

**Funding acquisition:** Shen-yu Zhu.

**Investigation:** Yuan-Chao Cao, Zhi-Xian Tang, Shen-yu Zhu.

**Methodology:** Zhi-Xian Tang, Shen-yu Zhu.

**Project administration:** KaiYun Mao, Zhi-Xian Tang, Shen-yu Zhu.

**Resources:** KaiYun Mao, Mao-Yan Si, Zhi-Xian Tang, Shen-yu Zhu.

**Software:** Yuan-Chao Cao.

**Supervision:** KaiYun Mao, Ding-yu Rao.

**Validation:** KaiYun Mao, Ding-yu Rao.

**Visualization:** KaiYun Mao, Liang Gu.

**Writing – original draft:** KaiYun Mao, Liang Gu.

**Writing – review & editing:** KaiYun Mao.
